# Downregulation of Circular RNA circPSD3 Promotes Metastasis by Modulating FBXW7 Expression in Clear Cell Renal Cell Carcinoma

**DOI:** 10.1155/2022/5084631

**Published:** 2022-03-07

**Authors:** Xuexia Xie, Haomin Li, Chongqing Gao, Yiqi Lai, Junjie Liang, Zhiwei Chen, Zheng Chen, Baoli Heng, Nan Yao, Caiyong Lai

**Affiliations:** ^1^Department of Urology, The First Affiliated Hospital of Jinan University, Guangzhou, China; ^2^Department of Pathophysiology, School of Medicine, Jinan University, Guangzhou, China; ^3^University of South China, Hengyang, China; ^4^Department of Hepatological Surgery, The First Affiliated Hospital of Jinan University, Guangzhou, China; ^5^Yingde Center, Institute of Kidney Surgery, Jinan University, Guangdong, China; ^6^Department of Urology, People's Hospital of Yingde City, Yingde, China; ^7^Postdoctoral Mobile Station, The First Clinical Medical College of Jinan University, Guangzhou, China; ^8^Department of Urology, The Sixth Affiliated Hospital of Jinan University, Dongguan, China; ^9^Yang Xi General Hospital People's Hospital, Yangjiang, China

## Abstract

Circular RNAs (circRNAs), a novel class of noncoding RNAs, have been shown to play critical regulatory roles in clear cell renal cell carcinoma (ccRCC). Metastasis is the main contributor to the poor prognosis of patients with ccRCC. However, the role of circRNAs in ccRCC metastasis has not been fully elucidated. In this study, microarray and RNA-seq analyses revealed that circPSD3 (hsa_circ_0002111) was dramatically downregulated in ccRCC tissues compared to adjacent nontumor tissues. A qRT-PCR analysis performed on our ccRCC cohorts confirmed the downregulation of circPSD3 in ccRCC tissues and further suggested that a low level of circPSD3 expression was associated with tumor metastasis in patients with ccRCC. Based on the results of functional studies, circPSD3 significantly inhibited cell migration, invasion, and the epithelial-mesenchymal transition (EMT) in vitro and blocked pulmonary metastasis in vivo. Mechanistically, circPSD3 functioned as a competing endogenous RNA for microRNA 25-3p (miR-25-3p) to regulate F-box and WD repeat domain-containing 7 (FBXW7) expression. Further verification indicated that circPSD3 overexpression restrained an EMT-like phenotype in cells, while miR-25-3p partially rescued these effects. In summary, circPSD3 inhibits tumor metastasis by repressing the miR-25-3p/FBXW7-EMT axis and might be developed as a potential diagnostic and therapeutic target for ccRCC.

## 1. Introduction

Renal cell carcinoma (RCC) has the highest lethality rate among urological tumors [1], and its incidence and mortality rates are increasing yearly. Clear cell renal cell carcinoma (ccRCC) is the most common type of RCC, accounting for 70–75% of all RCC cases [2]. The ccRCC is insidious, and approximately 25–30% of patients with ccRCC already have distant metastasis at the first diagnosis [3]. Surgery and other treatments for ccRCC have evolved in recent years [4]. However, the therapeutic effects on metastatic ccRCC are far from satisfactory, and the 5-year survival rate is extremely low [5]. Hence, the mechanisms underlying ccRCC metastasis must be elucidated to promote the further development of novel diagnostic and therapeutic strategies.

Circular RNAs (circRNAs) are characterized by the absence of a 5′-cap and a 3′poly-A tail [6]. Thus, circRNAs are easily resistant to RNase R digestion and are highly stable. CircRNAs were initially considered transcriptional errors but were later found to be widespread with the development of high-throughput sequencing [7, 8]. Currently, circRNAs are known to take part in the development of a host of diseases, such as immune diseases, cardiovascular diseases, neurological diseases, and tumors [9]. The biological functions and roles of circRNAs have been reported in all kinds of different cancers, suggesting the potential of circRNAs as biomarkers and therapeutic targets [10, 11]. Numerous studies have reported various functions of circRNAs, which serve as microRNA (miRNA) sponges by directly binding miRNAs [12]. For example, circASAP is upregulated in hepatocellular carcinoma (HCC) and accelerates HCC cell metastasis by combining miR-326 and miR-532-5p, thereby regulating the expressions of MAPK1 and CSF-1 [13]. According to previous studies, circ-AKT3 inhibits ccRCC cell metastasis by competitively sponging miR-296-3p and upregulating E-cadherin [14]. Additionally, circRNAs work by interacting with proteins, and some circRNAs are even translated into proteins [15, 16]. Recently, mitochondria-specific circRNA (SCAR) was reported as a therapeutic target for NASH by reducing mitochondrial reactive oxygen species (MROS) output, fibroblast activation, and inflammatory response [17]. However, many mechanisms and functions of circRNAs in ccRCC remain unclear.

In this study, circPSD3 was downregulated in ccRCC tissues from two public circRNA datasets. Lower circPSD3 expression correlated with more ccRCC metastases. Gain-of-function assays revealed that circPSD3 suppresses the metastasis of ccRCC cells in vitro and in vivo. Mechanistic studies indicated that circPSD3 inhibits F-box and WD repeat domain-containing 7 (FBXW7) degradation by sponging miR-25-3p and guiding the epithelial-mesenchymal transition (EMT). Therefore, circPSD3 is a potential diagnostic and therapeutic target for ccRCC.

## 2. Materials and Methods

### 2.1. Patient Samples

Eighty-one paired primary ccRCC tumor tissues and adjacent nontumor tissues were obtained from patients who had undergone radical nephrectomy or partial nephrectomy without neoadjuvant chemotherapy or radiotherapy at the First Affiliated Hospital of Jinan University (Guangzhou, China). All the samples were independently identified by two pathologists and stored at −80°C until use. All the samples were approved by the Ethics Committee of the First Affiliated Hospital of Jinan University, and informed consent was obtained from each patient.

### 2.2. Cell Culture and Transfection

All the cell lines were purchased from the ATCC. Each cell line was cultured in a medium containing 10% FBS and 1% double antibody (100 U/mL of penicillin + 100 mg/mL of streptomycin) and was placed in an incubator containing 5% CO_2_ at 37°C. Routine tests were performed using Hoechst DNA staining to ensure no mycoplasma contamination every six months. CircPSD3 small interfering RNAs (siRNAs) were complexed by RiboBio (Guangzhou, China). miR-25-3p mimics/inhibitors were obtained from GenePharma (Suzhou, China), and Lipofectamine 3000 (Invitrogen, USA) was used to transfect cells. Cells were transfected with a circPSD3 overexpression lentivirus using polybrene, and then 2 *μ*g/mL of puromycin (GeneChem, China) was used for 1 week to establish stably transfected cells. These sequences are shown in Supplementary Table 1.

### 2.3. RNA Extraction and Quantitative Real-Time PCR

Total RNA was extracted using TRIzol reagent (Invitrogen, USA). RNA was extracted from the nucleus and cytoplasmic components using a PARIS kit (Thermo Scientific, USA). The cDNA was extracted using a reverse transcription kit (Vazyme, Nanjing, China). qRT-PCR was enforced with SYBR Green Real-Time PCR Master Mix (Vazyme, Nanjing, China). GAPDH or U6 was used as a standard control. The relative RNA expression levels were calculated using the 2^−ΔΔCT^ method. The specific primers used are shown in Supplementary Table 1.

### 2.4. Cell Proliferation Assays

Cells were adjusted to 4 × 10^4^ cells/mL, and 100 *μ*L/well was inoculated into a 96-well plate, followed by the culture at 37°C for 24 h. Ten microliters of CCK-8 solution (Dojindo, Kumamoto, Japan) was added, and the absorbance was measured at 450 nm. The proliferation capacity of cells is proportional to the absorbance of cells.

### 2.5. Transwell Assays

Transwell assays were performed in twenty-four-well chambers (Corning, USA) with or without Matrigel (BD Biosciences) to assess cell motility. ACHN or Caki-1 cells (8 × 10^4^ cells/well) were inoculated in the upper chamber. After incubation for 24 h–48 h, the cells were recorded and imaged under a microscope.

### 2.6. Wound Healing Assays

Cells were wounded using a 20-*μ*L pipette, and images were acquired after washing with PBS (0 h time point). A serum-free medium was used to culture the cells. After one day, images were captured again (24 h time point). The wound healing rate = area of 24 h wound/area of 0 h wound × 100%.

### 2.7. Fluorescence In Situ Hybridization (FISH)

Cy3-labeled probe circPSD3 and FAM-labeled miR-25-3p probes (Supplementary Table 1) were obtained from GenePharma (Suzhou, China). The probes were transfected into cells using the FISH Kit (GenePharma, China). Images were acquired using a confocal microscope (Carl Zeiss).

### 2.8. Luciferase Reporter Assays

Briefly, cells were inoculated into 96-well plates and cotransfected with the corresponding pSICHECK2 vector and miR-25-3p mimics. The luciferase activity of fireflies and Renilla was measured using a dual-luciferase reporter kit (Promega, WI, USA). The luciferase activity is shown as the ratio of firefly luciferase activity to sea kidney luciferase activity. Independent experiments were conducted in triplicate.

### 2.9. Actinomycin D Assays

ACHN and Caki-1 cells were cultured in 2 *μ*g/ml of actinomycin D (Sigma, USA) after inoculation for 24 h and converged at 0, 4, 8, 12, and 24 h. RNA stability was analyzed using qRT-PCR. Each experiment was performed in triplicate.

### 2.10. RNA Pull-Down Assays

In total, 1 × 10^7^ cells were lysed in 50 *μ*L of lysis buffer (Thermo Scientific, USA), and then, 10 *μ*g of the biotin-labeled probe was added and incubated for 1 h. Washed streptavidin magnetic beads (Invitrogen, USA) were added to each binding reaction and incubated for 1 h. The beads were briefly washed six times with RIP washing buffer. Finally, the obtained RNA was analyzed by qRT-PCR.

### 2.11. Animal Study

Four-to six-week-old male BALB/c nude mice were purchased from the Guangdong Medical Laboratory Animal Center. For the subcutaneous xenografting of tumors, 5 × 10^6^ cells were injected into the right flank of each mouse. Tumor growth was recorded weekly, and the tumor volume (*V*) was calculated as follows: *V* = (*W*^2^ × *L*)/2. In the lung metastasis model, 6 × 10^6^ cells were injected into the tail vein of nude mice. After 6 weeks, the nude mice with lung metastases were observed by IVIS, their lungs were removed, and the number of metastases was photographed and recorded. All the animal studies were regulated in conformity to the Jinan University Guidelines for Animal Care and Use.

### 2.12. Hematoxylin and Eosin (H&E) Staining and Immunohistochemistry (IHC)

Paraffin sections were deacylated in xylene and rehydrated with ethanol. The slices were subjected to microwave heating in EDTA to extract the antigens and then incubated with goat serum at 37°C for 1 h and the indicated primary antibody at 4°C overnight. Subsequently, a horseradish peroxidase detection system was applied to quantify indicator protein expression (DAKO, Glostrup, Denmark).

### 2.13. Western Blot Analysis

Radioimmunoprecipitation assay lysis buffer (RIPA, Beyotime, China) was added to each group of cells to fully lyse and extract the total protein from the cells. SDS-PAGE (8–15%) was performed using 20 *μ*g of protein per lane. The protein isolates were transferred to PVDF membranes (Millipore, USA) and sealed in 5% skim milk powder solution at room temperature for 1 h. Rabbit polyclonal antibodies against FBXW7 (Abcam, USA), vimentin (Cell Signaling Technology, USA), E-cadherin (Cell Signaling Technology, USA), N-cadherin (Cell Signaling Technology, USA), and GAPDH (Cell Signaling Technology, USA) were added, followed by incubation at 4°C overnight. An appropriate secondary antibody (1 : 5000; Cell Signaling Technology, USA) was incubated in a 37°C shaker for 1 h, and the proteins were visualized using ECL chemiluminescence (Millipore, Germany). Image Lab software was used for data analysis.

### 2.14. Bioinformatics Analysis

The GSE100186 dataset (comprising 4 tumors and matched adjacent normal tissues) from the Gene Expression Omnibus (GEO, https://ncbi.nlm.nih.gov/geo/) was analyzed. The dataset (comprising 3 tumors and matched adjacent normal tissues) was obtained from our previous study [18]. The circRNA screening criteria were as follows: log_2_FC > 2 or <−2 and *P* < 0.05.

### 2.15. Statistical Analysis

SPSS 26.0 and GraphPad Prism 8 software were used for statistical analysis. The measurement data were tested for normality and homogeneity of variance, and the data were expressed as mean ± SD. Student's *t*-test and one-way ANOVA were performed to evaluate significant differences between two or more groups. *P* < 0.05 indicated that the difference was statistically significant.

## 3. Results

### 3.1. The Downregulation and Characterization of circPSD3 in ccRCC

**Figure 1:**
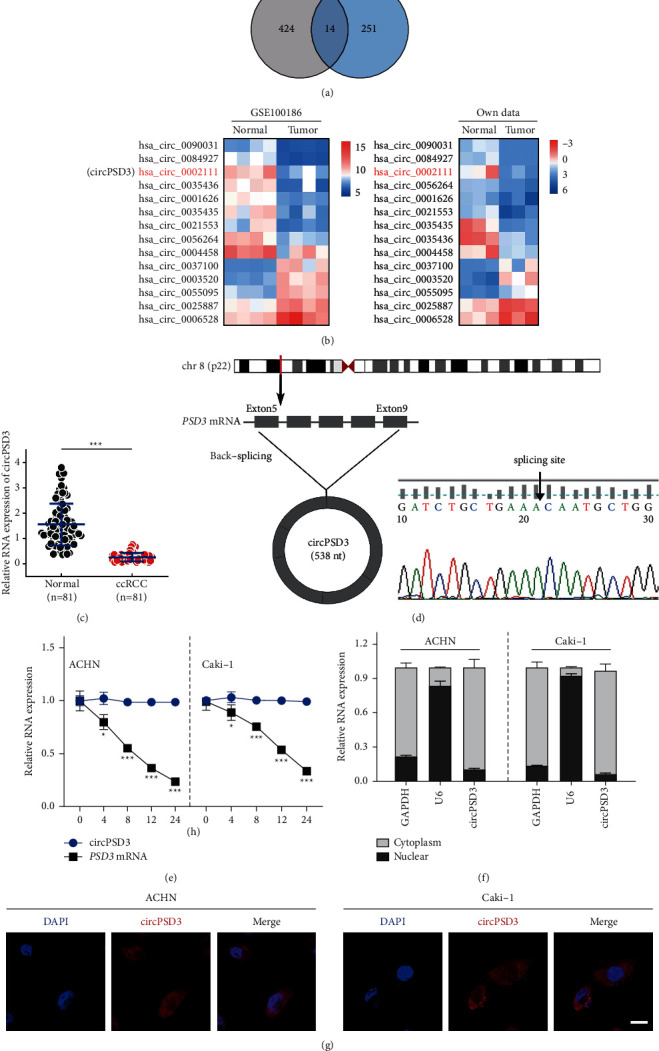
The downregulation and characterization of cirPSD3 in ccRCC. (a) Venn plot of the two datasets (GSE100186 and own data). Common circRNAs with a log_2_ (fold change) > 2 or <−2 and a *P*-value < 0.05 were selected. (b) Heatmaps of the two datasets. Red represents high expression, whereas blue represents low expression. (c) Analysis of the RNA levels of circPSD3 in an additional 81 paired samples of ccRCC by qRT-PCR. (d) The back-splice junction site of circPSD3 was identified by Sanger sequencing. (e) Analysis of the RNA abundance of circPSD3 and PSD3 in cells treated with actinomycin D (2 *μ*g/mL) at the indicated time points. *n* = 3 biologically independent samples. (f) RNA levels of circPSD3, GAPDH, and U6 in the nuclear and cytoplasmic fractions of ACHN and Caki-1 cells. (g) RNA FISH analysis of the cellular localization of circPSD3 in ACHN and Caki-1 cells. Nuclei were labeled with DAPI. Most of the circPSD3 is localized within the cytoplasm. Scale bar, 25 *μ*m. The data are presented as mean ± SD based on triplicate independent experiments.  ^*∗*^*P* < 0.05;  ^*∗*^ ^*∗*^ ^*∗*^*P* < 0.001.

First, we analyzed human ccRCC tissue samples from two datasets (GEO; GSE1000186 and own dataset) to examine the role of circRNAs in ccRCC advancement. Finally, 14 circRNAs that overlapped between the two datasets were extracted (Figure 1(a)), and heatmaps of their expression levels in the two datasets were plotted (Figure 1(b)). Significant downregulation of hsa_circ_0002111 (termed circPSD3) was observed in both cohorts; thus, circPSD3 was selected for further study. Next, circPSD3 was validated to have lower expression in ccRCC tissues than in normal renal tissues using qRT-PCR (Figure 1(c)).

The circPSD3 is derived from exons 5–9 of the PSD3 gene with a length of 538 nt. The back-splice junction site of circPSD3 was validated by Sanger sequencing. The identified sequence was the same as that in the circBase database (https://www.circbase.org/) (Figure 1(d)). We measured the half-life of circPSD3 in the indicated cells by treating them with actinomycin D to evaluate the stability of circPSD3, and circPSD3 was more stable than PSD3 mRNA (Figure 1(e)). We assessed circPSD3 expression in cells by qRT-PCR and found that circPSD3 was mainly distributed in the cytoplasm (Figure 1(f)). Similar results were obtained using the FISH assay (Figure 1(g)).

### 3.2. CircPSD3 Downregulation Is Associated with ccRCC Metastasis

**Figure 2:**
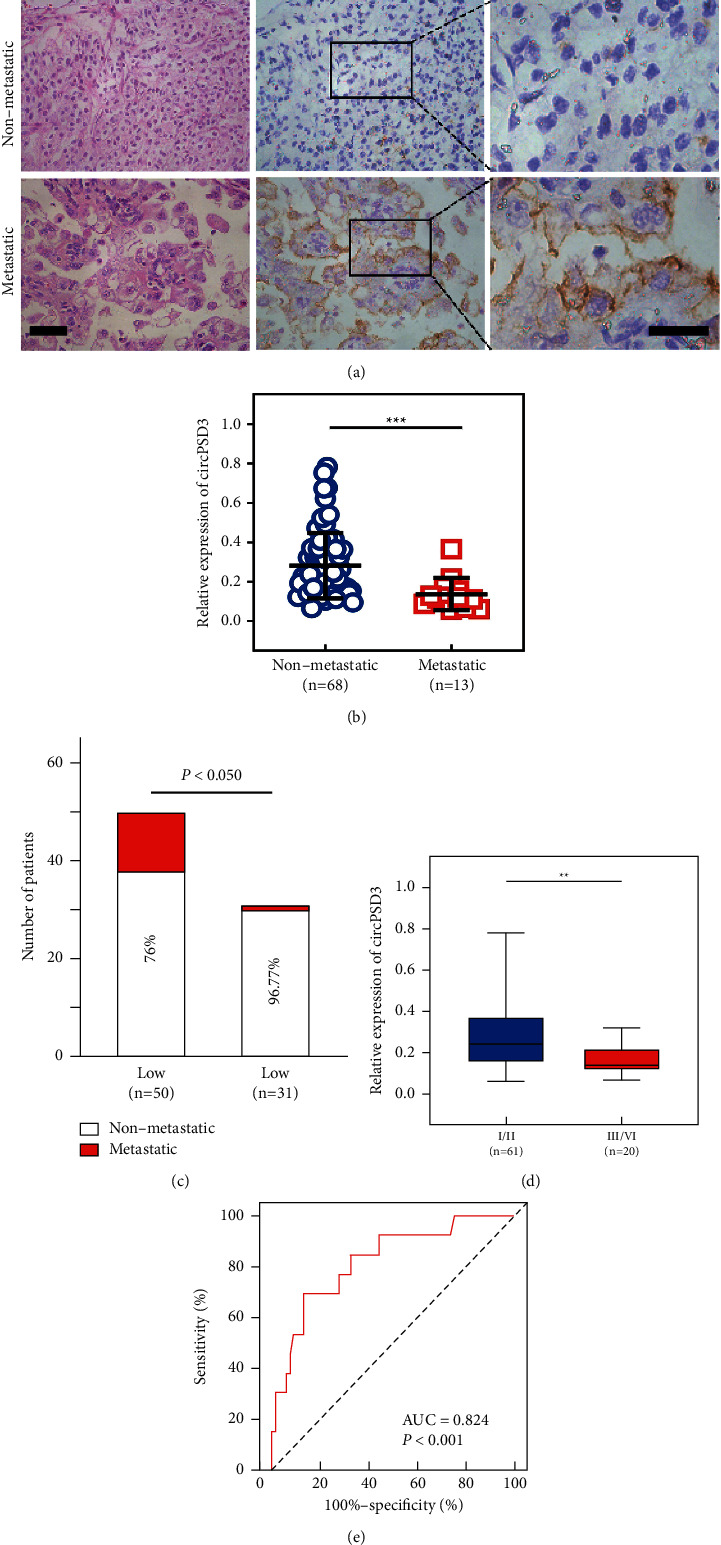
The downregulation of circPSD3 is associated with ccRCC metastasis. (a) H&E staining and IHC staining were performed to detect metastatic markers in ccRCC tissues. H&E: scale bar, 25 *μ*m; IHC: scale bar, 15 *μ*m. (b) The circPSD3 levels in the nonmetastatic and metastatic patients in our cohort were analyzed by qRT-PCR. (c) The bar chart shows the correlation between circPSD3 expression and metastasis in 81 human ccRCC tissue samples. (d) qRT-PCR analyses of circPSD3 expression in the Fuhrman stage III/IV group and Fuhrman stage I/II group. (e) ROC curve predicting metastasis in patients based on circPSD3 expression. The data are presented as the mean ± SD based on triplicate independent experiments.  ^*∗*^ ^*∗*^*P* < 0.01;  ^*∗*^ ^*∗*^ ^*∗*^*P* < 0.001.

Next, we analyzed the relationship between the circPSD3 levels and clinical characteristics and revealed that circPSD3 expression in ccRCC tissues was associated with metastasis (Table 1). The patients were diagnosed with metastatic and nonmetastatic ccRCC based on H&E staining and metastasis-related IHC markers (CD10) (Figure 2(a)). CD10 is considered a metastatic marker for ccRCC [19]. The levels of circPSD3 were significantly downregulated in metastatic ccRCC tissues compared with those in nonmetastatic ccRCC tissues (*P* < 0.001) (Figure 2(b)). Additionally, we analyzed the level of circPSD3 in 81 human metastatic and nonmetastatic specimens and showed that low circPSD3 expression was notably correlated with metastasis in ccRCC tissues (Figure 2(c)). Decreased levels of circPSD3 were dramatically associated with an advanced Fuhrman grade (Figure 2(d)). Next, we established a receiver operating characteristic (ROC) curve (68 tissues from patients without metastatic ccRCC were used as a control) and found that circPSD3 was highly correlated with ccRCC metastasis. The area under the ROC curve was 0.824 (*P* < 0.001) (Figure 2(e)). Furthermore, we investigated the expression of circPSD3 in ccRCC cell lines, revealing significant downregulation in cultured ccRCC cell lines (786O, 769P, A498, ACHN, and Caki-1) compared with a normal renal epithelial cell line (HK-2), especially in metastatic cell lines (ACHN and Caki-1) (Figure S1).

### 3.3. CircPSD3 Inhibits the Migration and Invasion of ccRCC Cells

**Figure 3:**
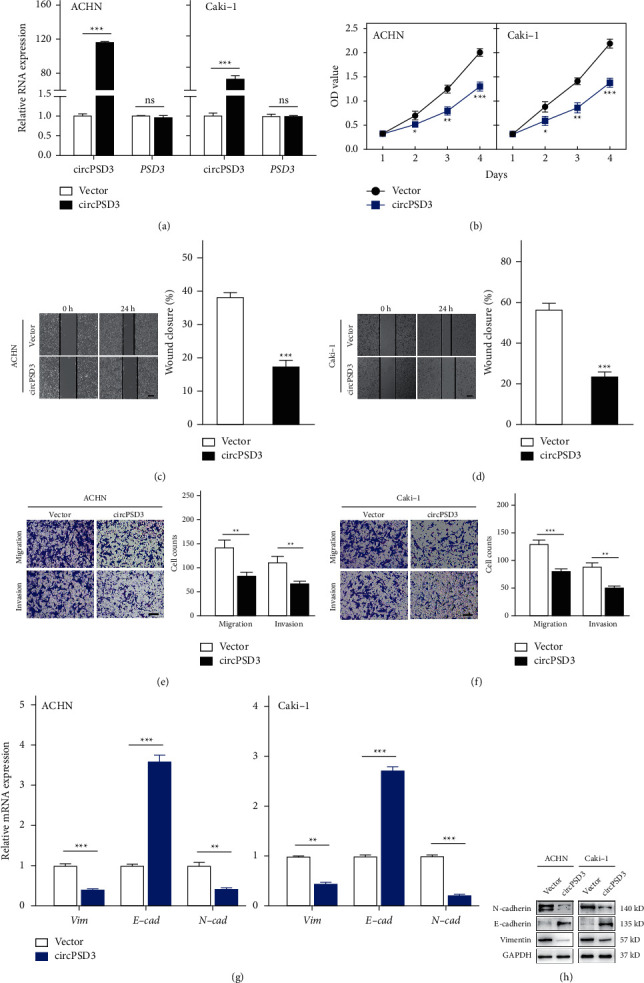
Overexpression of circPSD3 inhibits the migration and invasion of ccRCC cells. (a) qRT-PCR analysis of circPSD3 and PSD3 RNA expressions in ACHN and Caki-1 cells infected with a circPSD3 overexpression of lentivirus or vector control lentivirus. (b) Cell Counting Kit-8 assay of the effect of circPSD3 on the survival of the indicated ccRCC cells with stable circPSD3 overexpression. (c, d) Wound healing assays were performed to analyze the migration capacity of the indicated ccRCC cells. The dashed lines indicate the edge of migrating cells. Scale bars, 200 *μ*m. (e, f) Transwell assays were performed using Matrigel-coated or uncoated inserts to determine the migration and invasion capacities of the indicated ccRCC cells. Scale bar, 100 *μ*m. (g, h) qRT-PCR and Western blot analyses of the mRNA and protein levels of E-cadherin, N-cadherin, and vimentin in the indicated ccRCC cells. GAPDH was used as a loading control. The data are presented as mean ± SD based on triplicate independent experiments.  ^*∗*^*P* < 0.05;  ^*∗*^ ^*∗*^*P* < 0.01; and  ^*∗*^ ^*∗*^ ^*∗*^*P* < 0.001; and NS, no significance.

We used a lentivirus to overexpress circPSD3 in ACHN and Caki-1 cells and explored the functions of circPSD3 in ccRCC cells. In addition, we knocked down circPSD3 in ACHN and Caki-1 cells via transfecting a circPSD3-specific siRNA. The expression levels of circPSD3 and PSD3 in the cells with circPSD3 overexpression or knockdown were validated by qRT-PCR (Figures 3(a) and S2A). The CCK-8, scratch wound healing, and Transwell assays revealed that the proliferation, migration, and invasion abilities of ACHN and Caki-1 cells with circPSD3 overexpression were dramatically inhibited compared with those of the control groups (Figures 3(b)–3(f)). By contrast, knocking down circPSD3 promoted the malignant functions of ccRCC cells (Figures S2B–S2D).

EMT is a biological process in which tumor cells acquire the ability to migrate and invade. Therefore, we examined the relationship between circPSD3 and EMT. Notably, after upregulating the circPSD3 levels, the mRNA level of the epithelial marker E-cadherin was reregulated, and those of the mesenchymal markers, namely, N-cadherin and vimentin, were decreased (Figure 3(g)). We observed similar findings by Western blotting (Figure 3(h)).

### 3.4. CircPSD3 Inhibits ccRCC Metastasis by Mediating the EMT In Vivo

**Figure 4:**
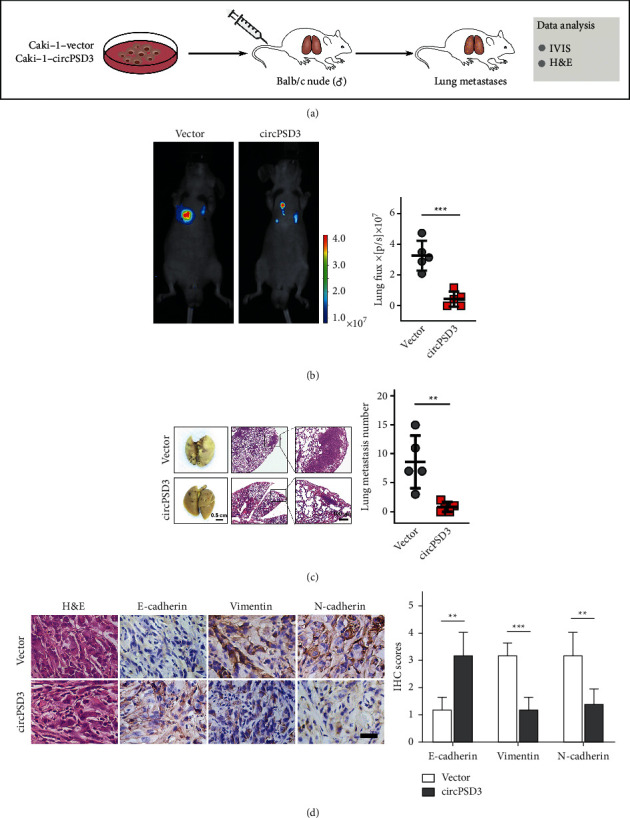
Upregulated circPSD3 inhibits ccRCC metastasis by mediating EMT in vivo. (a) Schematic of the lung metastasis model was established in BALB/C nude mice with the indicated cells. (b) Bioluminescence imaging and quantification of lung metastasis after injecting tumor cells into the tail veins of nude mice (*N* = 5). (c) Representative images of lung metastatic tumors were obtained from an in vivo lung metastasis model established via tail vein injection of tumor cells. The right panel shows the counts of the lung metastatic nodules (*N* = 5). (d) Representative images of H&E and IHC staining of E-cadherin, N-cadherin, and vimentin in xenograft tumors. Scale bar, 100 *μ*m. The data are presented as mean ± SD based on triplicate independent experiments.  ^*∗*^ ^*∗*^*P* < 0.01;  ^*∗*^ ^*∗*^ ^*∗*^*P* < 0.001.

To evaluate the biological function of circPSD3 that affects metastasis in vivo, we established a lung metastasis model by injecting circPSD3-overexpressing cells (Figure 4(a)). The in vivo imaging system (IVIS) and H&E staining showed that mice with circPSD3 overexpression had significantly fewer lung metastases than the controls (Figures 4(b)–4(c)). Subsequently, we built a subcutaneous transplant model with circPSD3-overexpressing Caki-1 cells. The circPSD3 overexpression dramatically inhibited the growth of xenograft tumors (Figure S3A) and resulted in an apparent decrease in the xenograft weight (Figure S3B). Next, the xenograft tumors were sectioned to determine whether the alteration in circPSD3 expression also influenced EMT in vivo. The IHC results showed that the changes in the EMT markers were consistent with the in vitro findings described above (Figure 4(d)). Based on these findings, circPSD3 plays an essential role in regulating EMT in ccRCC.

### 3.5. CircPSD3 Is a Sponge for miR-25-3p

**Figure 5:**
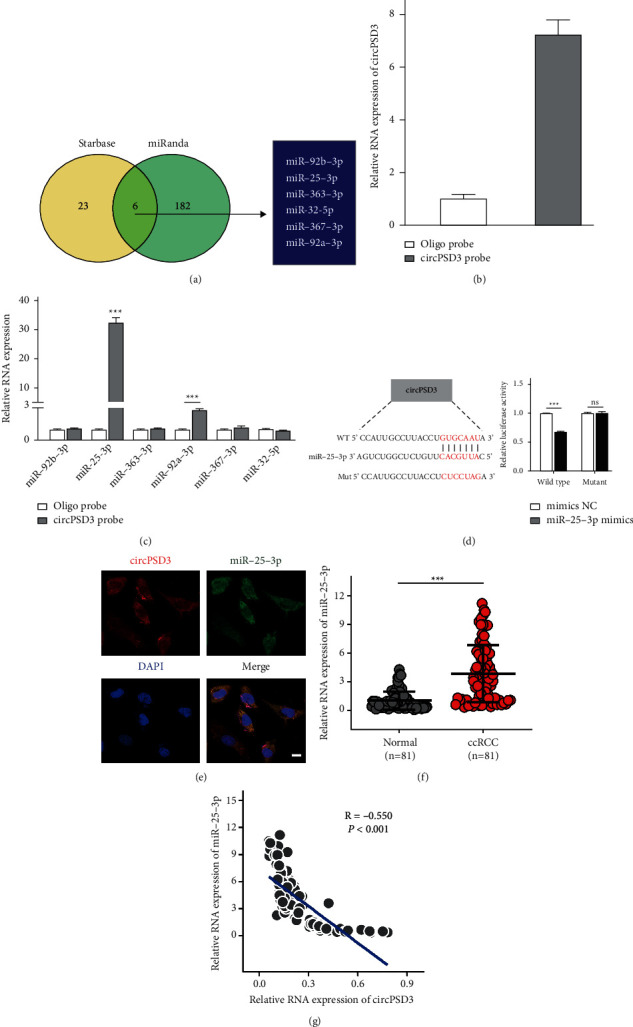
circPSD3 is a sponge for miR-25-3p. (a) Six candidate target miRNAs of circPSD3 were simultaneously predicted by miRanda and starBase software. (b, c) The circRNA pull-down experiments were performed with Caki-1 cells using a circPSD3-specific probe. The enrichment of circPSD3 and the 6 miRNAs was detected by qRT-PCR and normalized to the levels detected by a control probe. (d) The luciferase reporter assay was performed to detect the activity of luc-circPSD3 and luc-circPSD3 mutant in 293T cells after the transfection of miR-25-3p mimics. (e) RNA FISH immunofluorescence shows the colocalization of circPSD3 (red) with miR-25-3p (green) in Caki-1 cells. Scale bar, 50 *μ*m. (f) qRT-PCR was performed to detect miR-25-3p expression in adjacent nontumor and ccRCC tissues. (g) The circPSD3 expression was negatively correlated with miR-25-3p expression in our patient cohort (*n* = 81). The data are presented as mean ± SD.  ^*∗*^ ^*∗*^ ^*∗*^*P* < 0.001; NS, no significance.

CircRNAs located in the cytoplasm function as miRNA sponges. Thus, we used starBase and miRanda target prediction tools to identify miRNAs that might bind to circPSD3. In total, 6 miRNAs (miR-25-3p, miR-92b-3p, miR-363-3p, miR-32-5p, miR-367-3p, and miR-92a-3p) were taken for possible targets in the two databases (Figure 5(a)). RNA pull-down assays were conducted to appraise the mutual effects between these miRNAs and circPSD3. The efficiency of the biotin-labeled circPSD3 probe was performed by qRT-PCR (Figure 5(b)). Next, we extracted the circPSD3-specific pull-down miRNAs. The miR-25-3p and miR-92a-3 were highly expressed in the pull-down sample compared with those in the control, suggesting their association with circPSD3 in ccRCC cells (Figure 5(c)). According to the data from TCGA (starBase v3.0 project), miR-25-3p was abnormally upregulated in ccRCC tissues relative to normal tissues, while miR-92a-3p expression was not significantly different (*P*=0.27) between ccRCC tissues (*n* = 517) and normal tissues (*n* = 71) (Supplementary Figure 4). Thus, miR-25-3p is a potential target of circPSD3 in ccRCC cells. We performed the luciferase assay revealing that miR-25-3p interacts with circPSD3 (Figure 5(d)). FISH also identified that circPSD3 colocalized with miR-25-3p in the cytoplasm (Figure 5(e)). Additionally, we measured the miR-25-3p levels in our 81 paired ccRCC specimens by qRT-PCR. Indeed, miR-25-3p was dramatically upregulated in tumor tissues relative to adjacent normal renal samples, and the expressions of circPSD3 and miR-25-3p were negatively correlated (*P*-value < 0.001 and r-value of −0.55) in our ccRCC cohort (Figures 5(f) and 5(g)). These findings conformed to the competing endogenous RNA (ceRNA) mechanism of circRNAs and miRNAs. Overall, our results showed that circPSD3 functions as a sponge for miR-25-3p.

### 3.6. miR-25-3p Promotes the Metastasis of ccRCC by Targeting FBXW7

**Figure 6:**
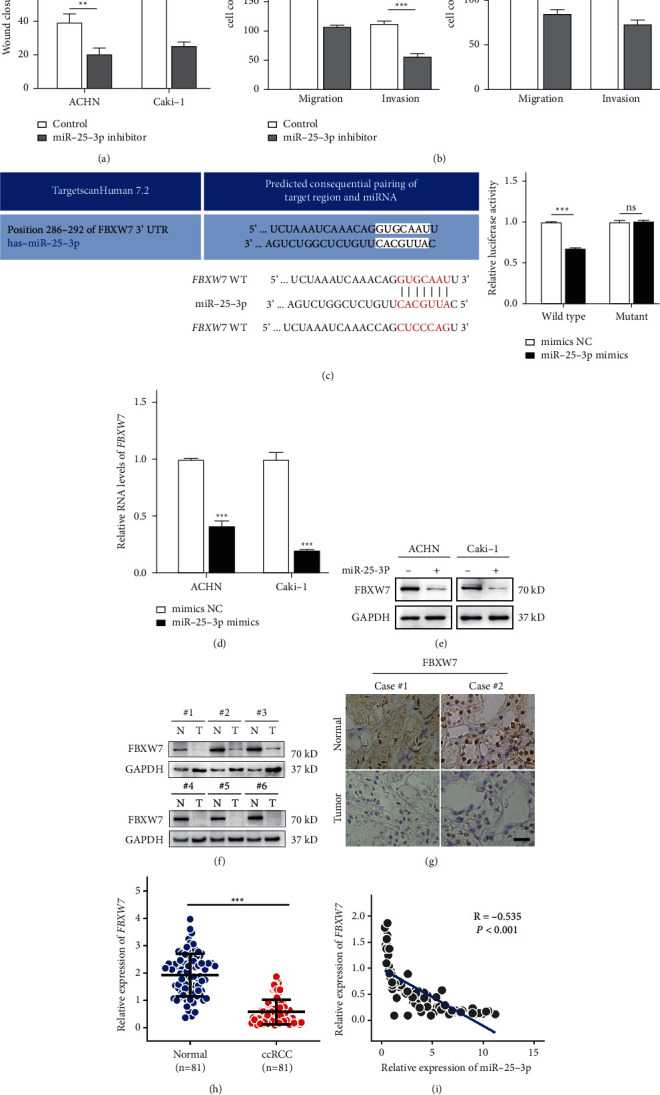
miR-25-3p promotes the metastasis of ccRCC through targeting FBXW7. (a) Wound healing assays were performed to analyze the migration capacity of ACHN and Caki-1 cells after silencing miR-25-3p. (b) Transwell assays were performed using Matrigel-coated or uncoated inserts to determine the migration and invasion capacities of the indicated cells. (c) The luciferase activity in 293T cells was analyzed after cotransfection with miR-25-3p mimics, miR-NC, and the WT or mutant luciferase reporter vectors (FBXW7). (d, e) qRT-PCR and Western blotting assays were performed to analyze the expression of FBXW7 in the indicated cells after treatment with the miR-25-3p mimic or NC. (f, g) Western blotting and representative IHC images of FBXW7 expression in ccRCC tissues and normal adjacent tissues. Scale bar, 50 *μ*m. (h) The relative expression levels of FBXW7 in ccRCC tissues and adjacent normal tissues were determined by qRT-PCR. (i) FBXW7 expression was negatively correlated with miR-25-3p expression in our patient cohort (*n* = 81). The data are presented as mean ± SD based on triplicate independent experiments.  ^*∗*^*P* < 0.05;  ^*∗*^ ^*∗*^*P* < 0.01;  ^*∗*^ ^*∗*^ ^*∗*^*P* < 0.001; and NS no significance.

The function of miR-25-3p in ccRCC remains unclear. The qRT-PCR analysis found that miR-25-3p was more highly expressed in all ccRCC cell lines than in human normal kidney epithelial cells (Figure S5A). These loss- and gain-of-function tests revealed that silencing miR-25-3p markedly inhibited the proliferation, migration, and invasion of Caki-1 and ACHN cells, while miR-25-3p upregulation produced reverse results (Figures 6(a), 6(b) and Figures 5SB–5SE).

Notably, miRNAs can target mRNAs. FBXW7 was already reported as a miR-25-3p target in glioblastoma [20]. Moreover, according to previous reports, FBXW7 inhibits the metastasis of ccRCC [21, 22]. As shown above, circPSD3 and miR-25-3p were linked with metastatic phenotypes. Thus, FBXW7 is a potential target of miR-25-3p in ccRCC cells. Next, we first identified the binding sites of miR-25-3p and FBXW7 using the TargetScanHuman website and then performed a luciferase reporter assay, which showed that the relative luciferase activity of the FBXW7 wild-type group was decreased by the miR-25-3p mimics relative to that of the FBXW7 mutant group (Figure 6(c)). qRT-PCR and Western blotting showed that the mRNA and protein levels of FBXW7 were decreased or increased after transfection with the miR-25-3p mimics or inhibitor (Figures 6(d), 6(e), 6SA, and 6SB).

Additionally, Western blotting and IHC showed lower FBXW7 expression in ccRCC tissues (Figures 6(f) and 6(g)). Our clinical ccRCC samples exhibited lower FBXW7 levels in ccRCC tissues than in control tissues, and miR-25-3p expression was inversely linked with FBXW7 expression in our ccRCC cohort (Figures 6(h) and 6(i)). Taken together, our results demonstrated that miR-25-3p promotes the metastasis of ccRCC by targeting FBXW7.

### 3.7. CircPSD3 Inhibits the EMT Process of ccRCC by Regulating miR-25-3p/FBXW7 Signaling

**Figure 7:**
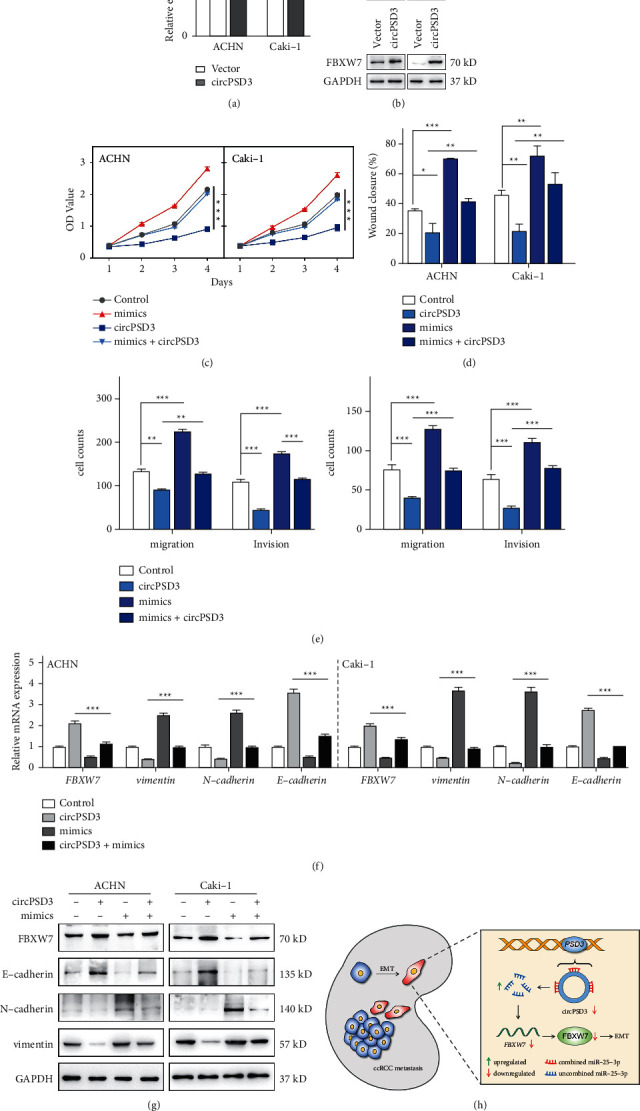
circPSD3 inhibits the EMT process in ccRCC cells by regulating miR-25-3p/FBXW7 signaling. (a, b) qRT-PCR and Western blotting were performed to detect the mRNA and protein levels of FBXW7, respectively, in ACHN and Caki-1 cells with stable circPSD3 overexpression. (c) CCK-8 assays were performed to analyze the viability of ACHN and Caki-1 cells after circPSD3 overexpression or transfection of the miR-25-3p mimics alone or in combination. (d, e) Wound healing and Transwell assays were performed to analyze the migration and invasion capacities of the indicated cells. (f, g) qRT-PCR and Western blotting were performed to detect the mRNA and protein levels, respectively, of FBXW7, E-cadherin, N-cadherin, and vimentin in the indicated cells. (h) Schematic graph illustrating that the circPSD3/miR-25-3p/FBXW7-EMT axis regulates ccRCC cell metastasis. The data are presented as mean ± SD based on triplicate independent experiments.  ^*∗*^*P* < 0.05;  ^*∗*^ ^*∗*^*P* < 0.01; and  ^*∗*^ ^*∗*^ ^*∗*^*P* < 0.001.

Rescue experiments were performed to assess whether circPSD3 inhibits metastasis by the miR-25-3p/FBXW7 axis. FBXW7 was strikingly upregulated in circPSD3-overexpressing ccRCC cells (Figures 7(a) and 7(b)). Functionally, the CCK-8, scratch wound healing, Transwell migration, and invasion assays showed that miR-25-3p promoted the metastasis of ccRCC cells, and this effect was blocked by circPSD3 overexpression (Figures 7(c)–7(e)). Additionally, the mRNA expression of FBXW77 was downregulated and positively related to circPSD3 expression in ccRCC tissues (Supplementary Figure 5F). Our previous study confirmed that circPSD3 blocked EMT activation, and related reports also documented that FBXW7 regulates EMT in renal cell carcinoma [21, 22]. Thus, we wondered whether circPSD3 affects EMT by regulating miR-25-3p/FBXW7. We gauged the expression levels of the same EMT pathway-related genes using qRT-PCR and Western blotting and found that circPSD3 affects EMT by regulating the miR-25-3p/FBXW7 levels (Figures 7(f) and 7(g)). Collectively, these observations demonstrate that circPSD3 inhibits ccRCC migration, invasion, and EMT by regulating miR-25-3p/FBXW7 signaling.

## 4. Discussion

Although circRNAs were considered to result from RNA splicing errors [23], the advancement of high-throughput sequencing techniques has led to the clear identification of various circRNAs that are stable and specifically expressed in mammalian cells [8]. Studies have revealed important roles for circRNAs in the tumorigenesis and progression of multiple tumor types, such as lung cancer [24], breast cancer [25], bladder cancer [26], HCC [27], and other types of carcinomas [28–30]. Nevertheless, the function of circRNAs in ccRCC, particularly in metastatic ccRCC, remains largely unknown. In this study, we screened the circRNAs that were differentially expressed by RNA-seq or microarrays. Following filtering, we identified a novel circRNA and circPSD3 and investigated their effect on ccRCC metastasis in vitro and in vivo.

The ceRNA hypothesis suggests that mRNAs and circRNAs share miRNA response elements, compete for the binding of miRNAs, and regulate the expression of each other, leading to the construction of a complex post-transcriptional regulatory network [31]. Most circRNAs in the cytoplasm contain one or more miRNA-binding elements [32], suggesting that they function as miRNA sponges to regulate the expression of downstream genes [33]. Notably, circPSD3 is more highly expressed in the cytoplasm of ccRCC cells. Therefore, we first explored the ability of circPSD3 to act as a miRNA sponge in ccRCC. In this study, we used bioinformatics analysis, luciferase reporter assays, and biotin pull-down assays to testify miR-25-3p as a target of circPSD3 in ccRCC cells. On the side, miR-25-3p expression was high and negatively correlated with that of circPSD3 in ccRCC tissues. According to previous studies, miR-25-3p facilitates the development of multiple tumors, including colorectal cancer [34], breast cancer [35], and pancreatic cancer [36]. However, the functions of miR-25-3p in ccRCC have not been reported. In our study, we confirmed that miR-25-3p promoted the migration and invasion of ccRCC cells, and this phenomenon was reversed by circPSD3.

FBXW7, a critical suppressor of human cancers [37], is a direct target of miR-25-3p, as determined by bioinformatics analysis and the luciferase reporter assay. Previous studies have indicated that the downregulation of FBXW7 facilitates tumor cell migration and invasion through inducing EMT in RCC [21, 22]. Consistent with previous studies, we discovered that FBXW7 was downregulated in our ccRCC tissues. Furthermore, FBXW7 mediated the ability of circPSD3 to suppress ccRCC cell migration and invasion activities that are regulated by miR-25-3p. EMT is the transformation of epithelial phenotypic cells into mesenchymal phenotypic cells [38, 39]. In cancer, EMT is the process by which epithelial-derived tumor cells acquire the ability to migrate and invade [40, 41]. Some studies have reported that circRNAs are associated with EMT [42]. In our study, circPSD3 was closely linked to EMT and inhibited cell invasion and metastasis through the miR-25-3p/FBXW7 axis.

In this study, we probed the expression and biological function of circPSD3 in ccRCC tissues and cells. However, we did not estimate circPSD3 expression in the blood serum, urine, or vesicles of patients with ccRCC because of the lack of specimens. Further studies are needed to explore the level of circPSD3 in ccRCC liquid specimens. Additionally, research on the molecular mechanism of circRNAs, including our study, has focused on miRNA sponges because of the cytoplasmic localization of circRNAs. However, this effect cannot be generally applied. Some studies have demonstrated that circRNAs directly interact with proteins, although they are mainly distributed in the cytoplasm. The circRNA-SORE directly binds to the oncogenic protein YBX1 to mediate sorafenib resistance in the cytoplasm of HCC [43]. Therefore, circPSD3 may directly bind some specific proteins in ccRCC cells. Inspired by these findings, we plan to study the other mechanisms of circPSD3 in the future.

## 5. Conclusions

In conclusion, our study provides the first evidence that circPSD3 is downregulated in patients with ccRCC and inhibits ccRCC cell metastasis by modulating EMT in vivo and in vitro. Mechanistically, the circPSD3/miR-25-3p/FBXW7 axis inhibits the EMT and metastasis of ccRCC cells (Figure 7(h)). Therefore, circPSD3 may be a potential diagnostic and therapeutic target in patients with ccRCC metastasis.

## Figures and Tables

**Table 1:** Association of circPSD3 expression with clinicopathological characteristics in ccRCC patients.

Characteristics	*n*	CircPSD3 expression		*P* value
		Low	High	
Age				
<60	41	25 (60.98%)	16 (39.03%)	0.888
≥ 60	40	25 (62.50%)	15 (37.50%)	
Gender				
Male	50	29 (58.00%)	21 (42.00%)	0.381
Female	31	21 (67.74%)	10 (32.26%)	
Tumor size (cm)				
≤ 4	47	26 (55.32%)	21 (44.68%)	0.163
> 4	34	24 (70.59%)	10 (29.41%)	
Fuhrman grade				
1 + 2	61	32 (52.46%)	29 (47.54%)	0.003 ^*∗*^
3 + 4	20	18 (90.00%)	2 (10.00%)	
T stage				
T1-T2	68	38 (55.88%)	30 (44.12%)	0.030 ^*∗*^
T3-T4	13	12 (92.31%)	1 (7.69%)	
Metastasis				
Yes	68	38 (55.88%)	30 (44.12%)	0.030 ^*∗*^
No	13	12 (92.31%)	1 (7.69%)	
Total	81	50	31	

^*∗*^*P* values are statistically significant.

## Data Availability

The data presented in this study are available on request from the corresponding author.

## Abbreviations

ccRCC: Clear cell renal cell carcinoma
circRNA: Circular RNA
miRNA: Micro-RNA
PSD3: Pleckstrin and SEC7 domain-containing 3
FBXW7: F-box and WD repeat domain-containing 7
EMT: Epithelial-mesenchymal transition
GEO: Gene expression omnibus
IHC: Immunohistochemistry.

## Supplementary Materials

Supplementary Materials
*Figure S1.* The expression of circPSD3 was downregulated in ccRCC cells. qRT-PCR analyses of circPSD3 expression in normal human kidney tubular epithelial cells (HK-2) and ccRCC cell lines (A498, 786O, 769P, Caki-1, and ACHN). The data are presented as the mean ± SD based on triplicate independent experiments. *Figure S2*. Silencing of circPSD3 promotes the migration and invasion of ccRCC cells. (A) qRT-PCR analysis of circPSD3 and PSD3 RNA expression in ACHN and Caki-1 cells transfected with the control siRNA or circPSD3 siRNA. (B) Cell Counting Kit-8 assays were performed on ACHN and ccRCC cells with circPSD3 knockdown. (C) Wound healing assays were performed to analyze the migration capacity of ACHN and Caki-1 cells with circPSD3 knockdown. (D) Transwell assays were performed with Matrigel-coated or uncoated inserts to determine the migration and invasion capacities of the indicated cells. The data are presented as the mean ± SD based on triplicate independent experiments.  ^*∗*^*P* < 0.05;  ^*∗*^ ^*∗*^*P* < 0.01;  ^*∗*^ ^*∗*^ ^*∗*^*P* < 0.001; and NS no significance. *Figure S3.* (A) Tumor volumes were measured and recorded every 3 days, and a growth curve was plotted. (B) Subcutaneous xenograft tumors generated with the indicated cells. The left panel shows the wet weights of the xenograft tumors. The data are presented as the mean ± SD based on triplicate independent experiments.  ^*∗*^ ^*∗*^ ^*∗*^*P* < 0.001. *Figure S4*. The RNA levels of miR-25-3p and miR-92a-3p in normal and ccRCC tissues (data from TCGA). *Figure S5*. (A) qRT-PCR assay was performed to detect miR-25-3p expression in human normal kidney tubular epithelial cells (HK-2) and ccRCC cell lines (786-O, ACHN, 769P, Caki-1, and A498). (B) Cell Counting Kit-8 assays were performed with ACHN and Caki-1 cells after silencing miR-25-3p. (C) Cell Counting Kit-8 assays were performed with the indicated cells after overexpressing miR-25-3p. (D) Wound healing assays were performed to analyze the migration capacities of the indicated cells after overexpressing miR-25-3p. (E) Transwell assays were performed with Matrigel-coated or uncoated inserts to determine the migration and invasion capacities of the indicated cells. (F) The expression of circPSD3 was positively correlated with FBXW7 expression in our patient cohort (*n* = 81). The data are presented as the mean ± SD based on triplicate independent experiments.  ^*∗*^*P* < 0.05;  ^*∗*^ ^*∗*^*P* < 0.01; and  ^*∗*^ ^*∗*^ ^*∗*^*P* < 0.001. *Figure S6*. Knockdown of miR-25-3p promotes FBXW7 expression. (A, B) qRT-PCR and Western blot assays were performed to analyze the expression of FBXW7 in the indicated cells after treatment with miR-25-3p inhibitors or the control. The data are presented as the mean ± SD.  ^*∗*^ ^*∗*^ ^*∗*^*P* < 0.001. *Table S1*. Primers and probes are listed.
