# Supplementary material for: Downregulation of Circular RNA circPSD3 Promotes Metastasis by Modulating FBXW7 Expression in Clear Cell Renal Cell Carcinoma
Source: J Oncol. 2022 Mar 7;2022:5084631. doi: 10.1155/2022/5084631 (PMC8920644; doi:10.1155/2022/5084631)
Supplement: Supplementary Materials — Figure S1. The expression of circPSD3 was downregulated in ccRCC cells. qRT-PCR analyses of circPSD3 expression in normal human kidney tubular epithelial cells (HK-2) and ccRCC cell lines (A498, 786O, 769P, Caki-1, and ACHN). The data are presented as the mean ± SD based on triplicate independent experiments. Figure S2. Silencing of circPSD3 promotes the migration and invasion of ccRCC cells. (A) qRT-PCR analysis of circPSD3 and PSD3 RNA expression in ACHN and Caki-1 cells transfected with the control siRNA or circPSD3 siRNA. (B) Cell Counting Kit-8 assays were performed on ACHN and ccRCC cells with circPSD3 knockdown. (C) Wound healing assays were performed to analyze the migration capacity of ACHN and Caki-1 cells with circPSD3 knockdown. (D) Transwell assays were performed with Matrigel-coated or uncoated inserts to determine the migration and invasion capacities of the indicated cells. The data are presented as the mean ± SD based on triplicate independent experiments. ∗P < 0.05; ∗ ∗P < 0.01; ∗ ∗ ∗P < 0.001; and NS no significance. Figure S3. (A) Tumor volumes were measured and recorded every 3 days, and a growth curve was plotted. (B) Subcutaneous xenograft tumors generated with the indicated cells. The left panel shows the wet weights of the xenograft tumors. The data are presented as the mean ± SD based on triplicate independent experiments. ∗ ∗ ∗P < 0.001. Figure S4. The RNA levels of miR-25-3p and miR-92a-3p in normal and ccRCC tissues (data from TCGA). Figure S5. (A) qRT-PCR assay was performed to detect miR-25-3p expression in human normal kidney tubular epithelial cells (HK-2) and ccRCC cell lines (786-O, ACHN, 769P, Caki-1, and A498). (B) Cell Counting Kit-8 assays were performed with ACHN and Caki-1 cells after silencing miR-25-3p. (C) Cell Counting Kit-8 assays were performed with the indicated cells after overexpressing miR-25-3p. (D) Wound healing assays were performed to analyze the migration capacities of the indicated cells after overexpressing m [file 5084631.f1.docx]

Supplementary Materials for

**Down-regulation of circular RNA circPSD3 promotes metastasis by modulating FBXW7 expression in clear cell renal cell carcinoma**

Xuexia Xie^1*^, Haomin Li^1*^, Chongqing Gao^3^, Yiqi Lai^4^, Junjie Liang^6^, Zhiwei Chen^6^, Zheng Chen^1^, Baoli Heng^7,8,9#^, Nan Yao^3#^, Caiyong Lai^1,2,5#^

^1^Department of Urology, The First Affiliated Hospital of Jinan University, Guangzhou, China.

^2^Department of Urology, The sixth Affiliated Hospital of Jinan University, Dongguan, China.

^3^Department of Pathophysiology, School of Medicine, Jinan University, Guangzhou, China.

^4^University of South China, Hengyang, China.

^5^Yang Xi General Hospital People’s Hospital, Yangjiang, China.

^6^Department of Hepatological Surgery, The First Affiliated Hospital of Jinan University, Guangzhou, China.

^7^Yingde Center, Institute of Kidney Surgery, Jinan University, Guangdong, China.

^8^Department of Urology, People’s Hospital of Yingde City, Yingde, China.

^9^Postdoctoral Mobile Station, the First Clinical Medical College of Jinan University, Guangzhou, China.

*These authors contributed equally to this work.

^#^ To whom correspondence should be addressed:

Dr. Caiyong Lai, Department of Urology, The sixth Affiliated Hospital of Jinan University, Guangdong, China; E-mail: lcy2015@jnu.edu.cn; Tel: +86-769-8350-8195.

Dr. Nan Yao, Department of Pathophysiology, School of Medicine, Jinan University, Guangzhou, China. E-mail: yaon107@jnu.edu.cn; Tel: +86-20-8522-0253.

Dr. Baoli Heng, Yingde Center, Institute of Kidney Surgery, Jinan University, Guangdong, China; E-mail: bolyheng@126.com; Tel: +86-20-3868-8303.

**This PDF file includes:**

Supplementary Figures. S1 to S6

Supplementary Table S1

**
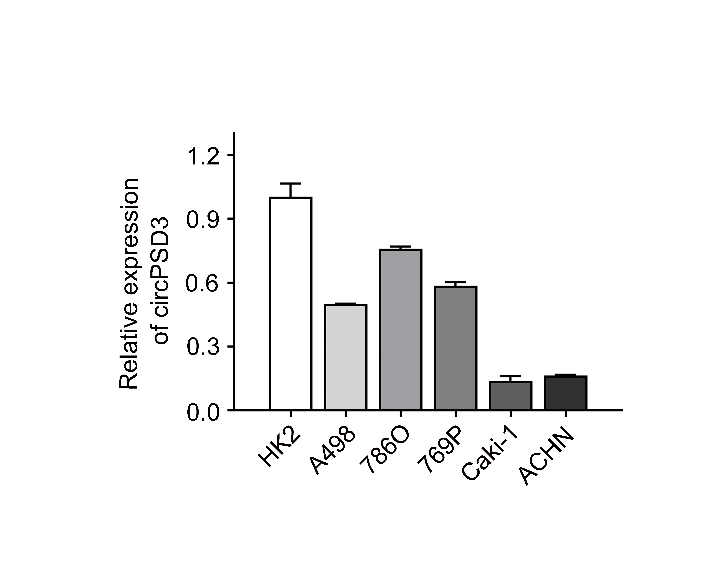
Supplementary Figures**

**Fig S1. The expression of circPSD3 was downregulated in ccRCC cells.** qRT-PCR analyses of circPSD3 expression in normal human kidney tubular epithelial cells (HK-2) and ccRCC cell lines (A498, 786O, 769P, Caki-1, and ACHN). The data are presented as the means ± SD based on triplicate independent experiments.

**
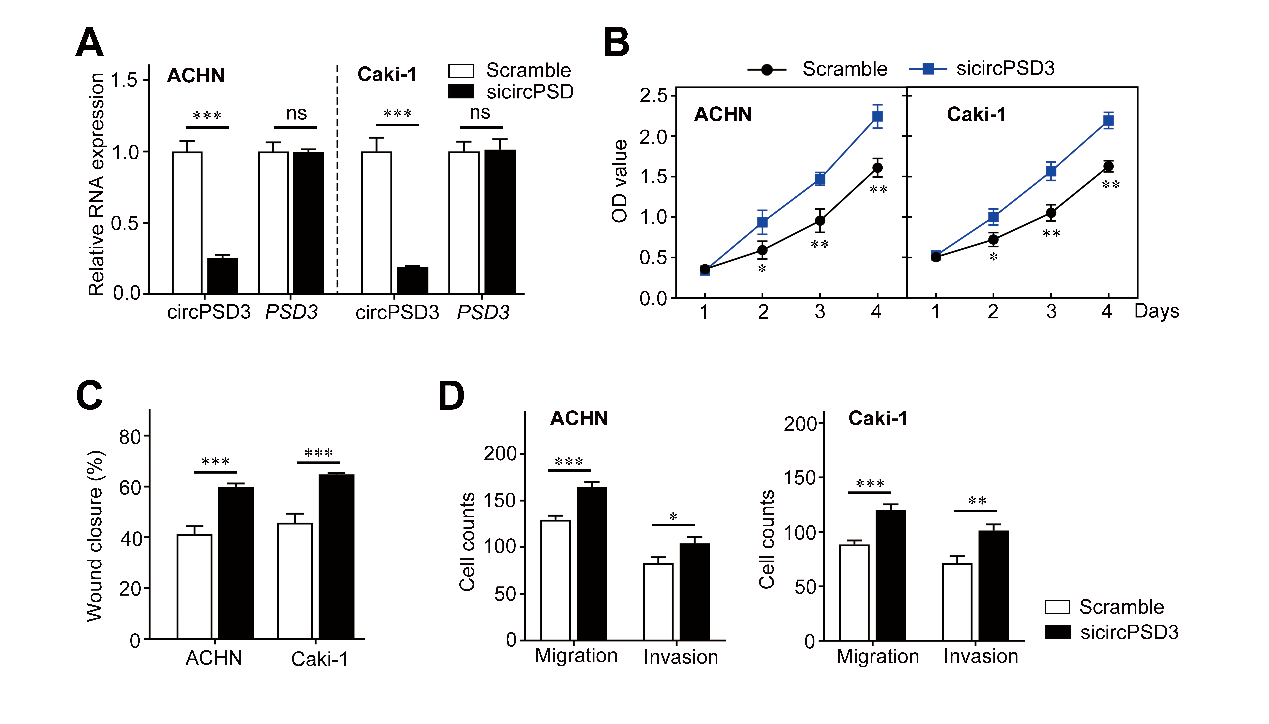
**

**Fig S2. Silencing of circPSD3 promotes the migration and invasion of ccRCC cells. (A)** qRT-PCR analysis of circPSD3 and PSD3 RNA expression in ACHN and Caki-1 cells transfected with the control siRNA or circPSD3 siRNA. **(B)** Cell Counting Kit-8 assays were performed on ACHN and ccRCC cells with circPSD3 knockdown. **(C)** Wound healing assays were performed to analyze the migration capacity of ACHN and Caki-1 cells with circPSD3 knockdown. **(D)** Transwell assays were performed with Matrigel-coated or uncoated inserts to determine the migration and invasion capacities of the indicated cells. The data are presented as the means ± SD based on triplicate independent experiments. *P < 0.05; **P < 0.01; ***P < 0.001; NS no significance.

**
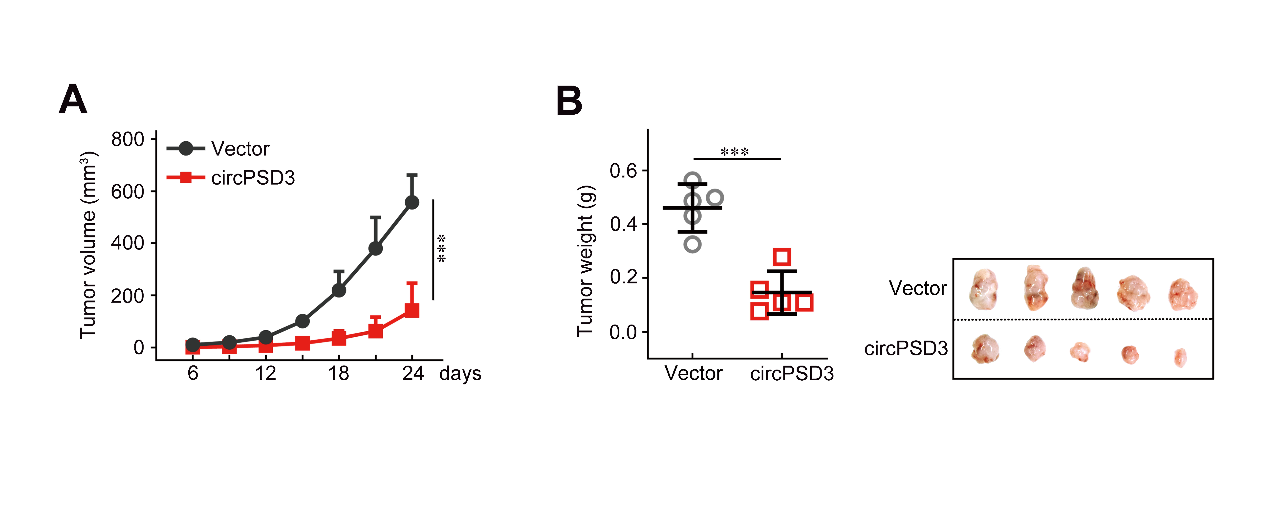
**

**Fig S3 (A)** Tumor volumes were measured and recorded every 3 days, and a growth curve was plotted. **(B)** Subcutaneous xenograft tumors generated with the indicated cells. The left panel shows the wet weights of the xenograft tumors. The data are presented as the means ± SD based on triplicate independent experiments. ***P < 0.001.

**
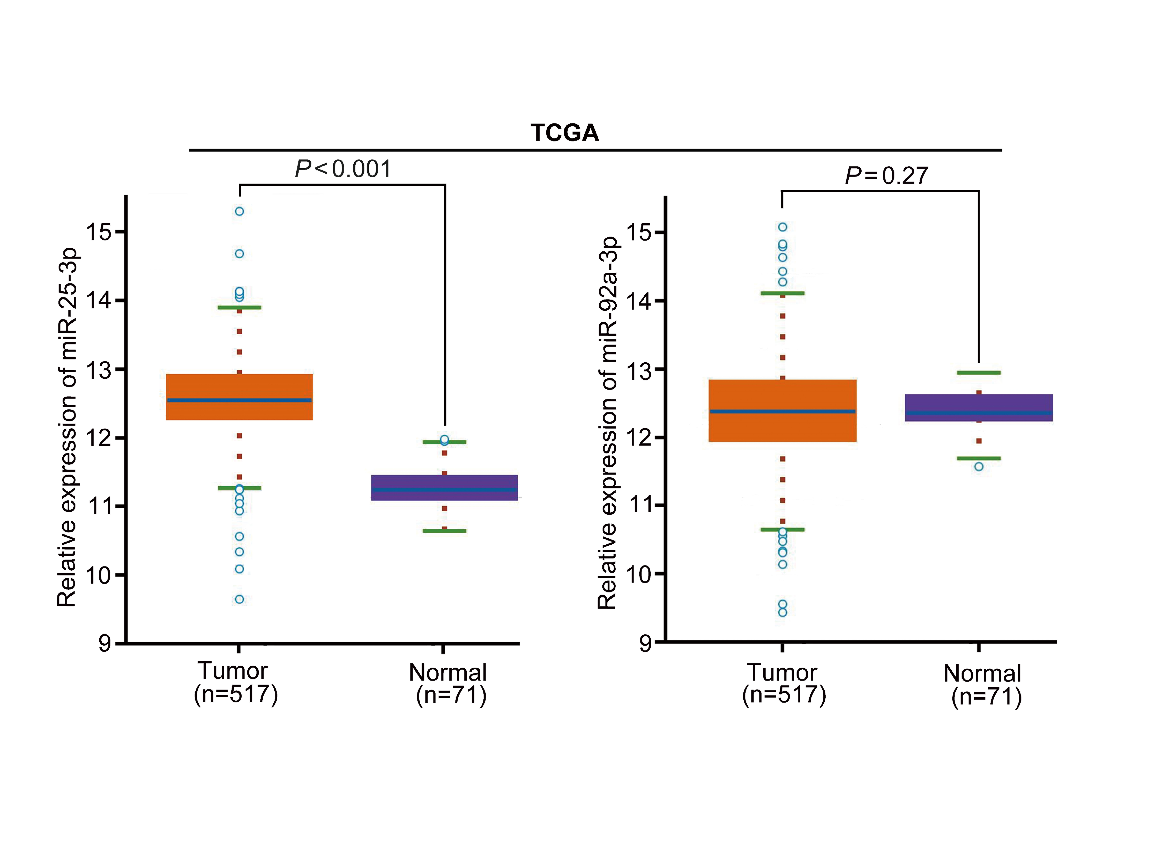
**

**Fig S4.** The RNA levels of miR-25-3p and miR-92a-3p in normal and ccRCC tissues (data from TCGA).

**
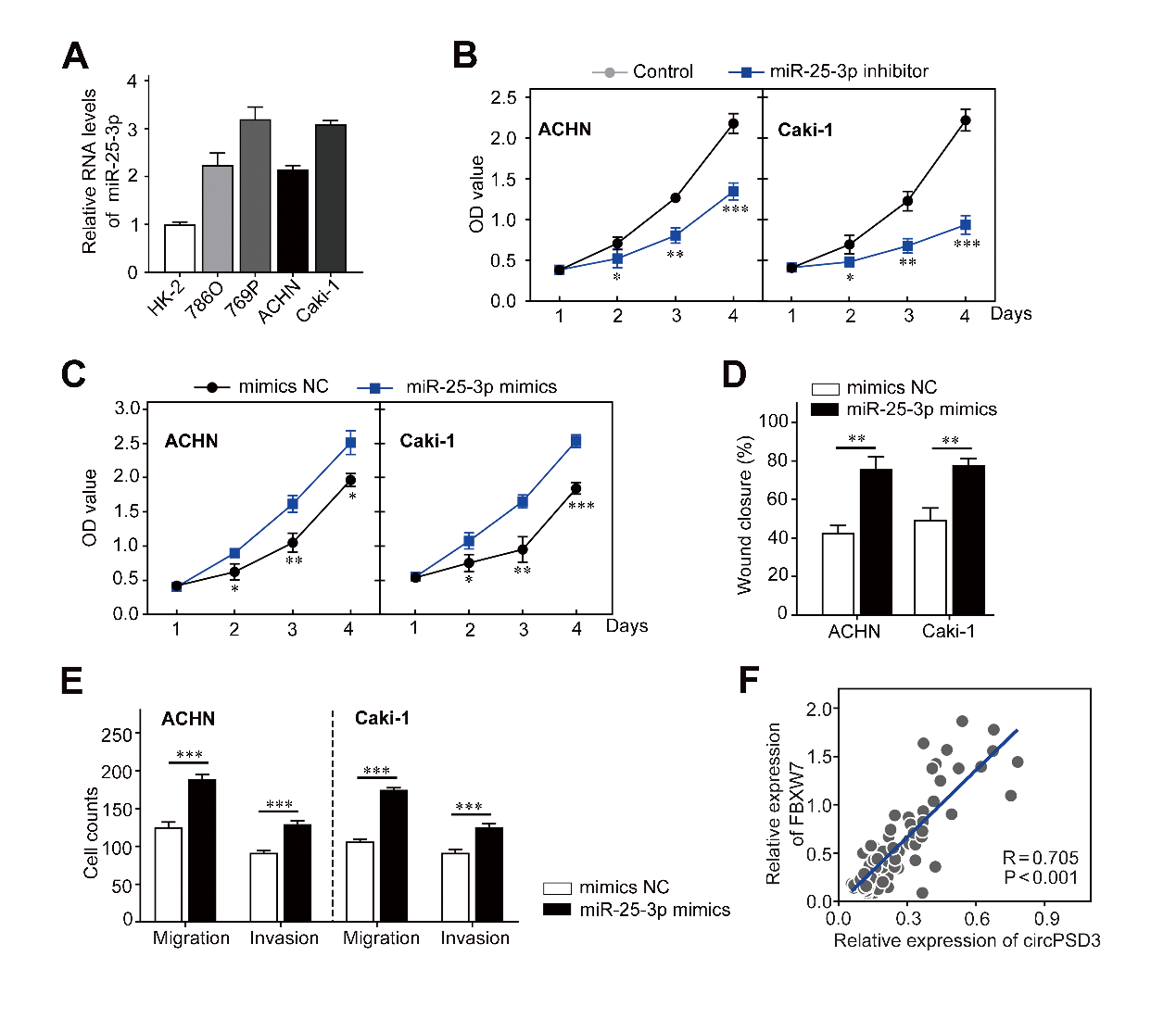
**

**Fig S5. (A)** qRT-PCR assay was performed to detect miR-25-3p expression in human normal kidney tubular epithelial cells (HK-2) and ccRCC cell lines (786-O, ACHN, 769P, Caki-1, and A498). **(B)** Cell Counting Kit-8 assays were performed with ACHN and Caki-1 cells after silencing miR-25-3p. **(C)** Cell Counting Kit-8 assays were performed with the indicated cells after overexpressing miR-25-3p. **(D)** Wound healing assays were performed to analyze the migration capacities of the indicated cells after overexpressing miR-25-3p. **(E)** Transwell assays were performed with Matrigel-coated or uncoated inserts to determine the migration and invasion capacities of the indicated cells. **(F)** The expression of circPSD3 was positively correlated with FBXW7 expression in our patient cohort (n = 81). The data are presented as the means ± SD based on triplicate independent experiments. *P < 0.05; **P < 0.01; ***P < 0.001.

**
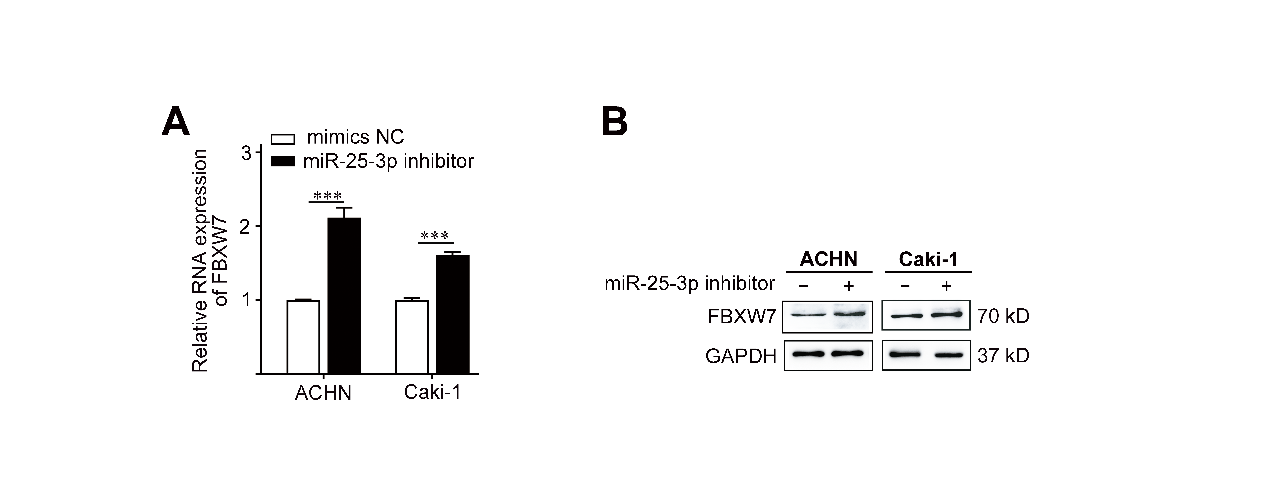
**

**Fig S6. Knockdown of miR-25-3p promotes FBXW7 expression. (A, B)** qRT-PCR and western blot assays were performed to analyze the expression of FBXW7 in the indicated cells after treatment with miR-25-3p inhibitors or the control. The data are presented as the means ± SD. ***P < 0.001.

| Gene | sequence (5′ to 3′) |
| --- | --- |
| CircPSD3 | Forward: TCTCCAAGGATCTGCTGAAACA |
|  | Reverse: GCTTCCACATTGCTGCTGGTA |
| PSD3 | Forward: GCCACGCCATCTCATCTCATCAG |
|  | Reverse: CTCCACTCTCATCAAGACGCACAG |
| GAPDH | Forward: GTCAAGGCTGAGAACGGGAA |
|  | Revers`e: AAATGAGCCCCAGCCTTCTC |
| U6 | Forward: CTCGCTTCGGCAGCACA |
|  | Reverse: AACGCTTCACGAATTTGCGT |
| FBXW7 | Forward: GCATCAGAGTGCTGTGACCT |
|  | Reverse: CCCCCACTCTCCAATGTGAC |
| E-cadherin | Forward: CGAGAGCTACACGTTCACGG |
|  | Reverse: GGGTGTCGAGGGAAAAATAGG |
| Vimentin | Forward: TGGCCGACGCCATCAACACC |
|  | Reverse: CACCTCGACGCGGGCTTTG |
| N-cadherin | Forward: GCGCGTGAAGGTTTGCCAGTG |
|  | Reverse: CCGGCGTTTCATCCATACCACAA |
| miR-92a-3p | Forward: TTGCACTTGTCCCGGCCTG |
| miR-92b-3p | Forward: GCACTCGTCCCGGCCTC |
| miR-25-3p | Forward: CATTGCACTTGTCTCGGTC |
| miR-32-5p | Forward: GGTATTGCACATTACTAAGTTGC |
| miR-363-3p | Forward: GAATTGCACGGTATCCATCTG |
| miR-367-3p | Forward: GAATTGCACTTTAGCAATGGTG |
| CircPSD3 FISH probe | GUUUUCA+CCCCAGCAUUG+UUUCAGCAGA+UCCUUGGAG |
| miR-25-3p FISH probe | UCAGA+CCGAGACAAG+UGCAAUG |
| CircPSD3 siRNA | GATCTGCTGAAACAATGCT |

**Table S1.Primers and probes are listed as follow:**
